# A unique dynamin-related protein is essential for mitochondrial fission in *Toxoplasma gondii*

**DOI:** 10.1371/journal.ppat.1007512

**Published:** 2019-04-04

**Authors:** Carmen Melatti, Manuela Pieperhoff, Leandro Lemgruber, Ehmke Pohl, Lilach Sheiner, Markus Meissner

**Affiliations:** 1 Wellcome Trust Centre for Molecular Parasitology, Institute of Infection, Immunity & Inflammation, Glasgow Biomedical Research Centre, University of Glasgow, Glasgow, United Kingdom; 2 Department of Biosciences, & Biophysical Sciences Institute, Durham University, Durham, United Kingdom; University of South Florida, UNITED STATES

## Abstract

The single mitochondrion of apicomplexan protozoa is thought to be critical for all stages of the life cycle, and is a validated drug target against these important human and veterinary parasites. In contrast to other eukaryotes, replication of the mitochondrion is tightly linked to the cell cycle. A key step in mitochondrial segregation is the fission event, which in many eukaryotes occurs by the action of dynamins constricting the outer membrane of the mitochondria from the cytosolic face. To date, none of the components of the apicomplexan fission machinery have been identified and validated. We identify here a highly divergent, dynamin-related protein (*Tg*DrpC), conserved in apicomplexans as essential for mitochondrial biogenesis and potentially for fission in *Toxoplasma gondii*. We show that *Tg*DrpC is found adjacent to the mitochondrion, and is localised both at its periphery and at its basal part, where fission is expected to occur. We demonstrate that depletion or dominant negative expression of *Tg*DrpC results in interconnected mitochondria and ultimately in drastic changes in mitochondrial morphology, as well as in parasite death. Intriguingly, we find that the canonical adaptor *Tg*Fis1 is not required for mitochondrial fission. The identification of an Apicomplexa-specific enzyme required for mitochondrial biogenesis and essential for parasite growth highlights parasite adaptation. This work paves the way for future drug development targeting *Tg*DrpC, and for the analysis of additional partners involved in this crucial step of apicomplexan multiplication.

## Author summary

Mitochondria fission is mediated by an elaborate “fission complex” of dynamin and receptor proteins, best studied in organisms such as yeast and mammalian cells. Little is known about this process in apicomplexan parasites, unicellular eukaryotes characterised by a single, lasso-shaped mitochondrion. Here, we analyse the conservation of the fission complex in *Toxoplasma gondii*; we find that the protein *Tg*Fis1 is the only mitochondrial adaptor that is highly conserved, but our characterisation shows that it is not essential for parasite growth or for mitochondrial dynamics. In contrast, we find that the dynamin-related protein C (*Tg*DrpC) is a key factor of mitochondrial biogenesis in *T*. *gondii*, showing that its recruitment to the basal part of the mitochondrion is essential for the formation of mature, separate organelles. Furthermore, our analysis shows that *Tg*DrpC GTPase domain is conserved and essential for its function, highlighting its potential as novel drug target.

## Introduction

Members of the Apicomplexa phylum are unicellular obligate parasites. Being eukaryotes, they share many features of common model organisms, such as a double-membrane nuclear envelope and a conserved endomembrane system. However, they are also set apart by various novel characteristics, such as the phylum-specific apical complex, which contains specialised secretory organelles; a pellicle-like membranous compartment called the inner membrane complex (IMC); and an atypical non-photosynthetic plastid, the apicoplast.

Moreover, they present a single, ramified mitochondrion whose genome is severely reduced, encoding only three proteins [1–4]; while this organelle is usually lasso-shaped in intracellular tachyzoites, in extracellular parasites its morphology is changed drastically, reducing its proximity to the parasite periphery [5]. The mitochondrion is thought to be essential for parasite survival at all stages, as it has a central function both in energy generation and in the production of precursors of other pathways, like pyrimidine and heme biosynthesis [6–9]. Thus, it is no surprise that the mitochondrion is a validated drug target for both *Plasmodium* spp. and *T*. *gondii*, as reviewed in [10, 11].

Nevertheless, our knowledge about the mechanisms controlling mitochondrial behaviour in Apicomplexa is limited. In most eukaryotes studied, mitochondria form a dynamic network, whose shape depends on the opposing mechanisms of fission, fusion and motility, which occur throughout the cell cycle [12–15]. Conversely, previous research in *T*. *gondii* has shown that the duplication of the mitochondrion in tachyzoites is tightly linked to cell division (known as endodyogeny in *T*. *gondii*), similar to the single-cell red alga *Cyanidioschyzon merolae* [15], and spontaneous fission of mitochondria in resting intracellular parasites is not clearly observed [5, 16]. *T*. *gondii* mitochondrial duplication starts at the early stages of daughter formation, when the IMC is beginning to form, at which point the mitochondrion branches in multiple locations: these elongations are not immediately incorporated in the developing daughter cells, but stay close to the periphery of the mother until the last stage of cytokinesis. When the daughters are almost fully formed, and start emerging from the mother cell, the mitochondria migrate into them. At this point, the new mitochondria are still linked to each other, with the interconnection seen at the base of the new daughter cells. These interconnections can remain present for a variable time, but it is assumed that they eventually get cleaved leading to individual mitochondria. The mechanism underlying this putative fission step is not yet characterised.

In all eukaryotes studied to date, the key player of mitochondrial fission is a dynamin-related protein, called Drp1 or Dnm1 in humans and yeast, respectively, and DRP3A/B in plants [17–19]. This mechanochemical enzyme, characterised by a large GTPase domain, is essential for the remodelling of the mitochondria membrane, and is typically recruited to the mitochondrial outer membrane (MOM) at ER/actin preconstricted fission sites [20, 21]. Recruitment is mediated by an adaptor complex consisting of receptor/adaptor proteins, whose composition varies significantly between different eukaryotes (Table 1) [22–26]. After its recruitment, the dynamin-related protein forms spirals around the membrane. Subsequent GTP hydrolysis provokes a conformational change that results in a two-fold decrease in the diameter of the spiral, and finally in the cleavage of the membrane [27–29]. In mammalian cells this last step requires an additional constriction mediated by the classical dynamin Dyn2 [30]. Thus, dynamin-related proteins are central to mitochondrial fission.

**Table 1:** known factors involved in mitochondrial fission in yeast, humans and plants and their conservation in *T*. *gondii* genome.

Protein name	Function	*Toxoplasma gondii* homolog
Drp1 (human)/Dnm1(yeast)/Drp3A-B(plants)	Dynamin related protein	TgDrpA (TGME49_267800), TgDrpB (TGME49_321620), TgDrpC (TGME49_270690)
Fis1 (human, yeast, plants)	Mitochondrial outer membrane adaptor	TgFis1 (TGME49_263323)
Mdv1/Caf4/Num1/Mdm36 (yeast)	Mitochondrial receptor for Dnm1	-
Mff (Human)	Recruitment of Drp1 to the mitochondrial membrane	-
MiD49/50 (Human)	Drp1 receptor	-
ELM1 (plants)	Recruitment of Drp3 to the mitochondrial membrane	-

Two well conserved dynamin-related proteins, *Tg*DrpA and *Tg*DrpB, were previously identified and functionally characterised in *T*. *gondii*, where they were shown to play essential roles in apicoplast division [31] and in the biogenesis of the unique secretory organelles [32], respectively. While a minor role for *Tg*DrpA in mitochondrial biogenesis could not be ruled out, no clear implication in mitochondrial division was found. Bioinformatics identified a third potential member of the dynamin superfamily in these parasites [31–33]: this protein was called *Tg*DrpC. Analysis of the phylogenetic distribution of *Tg*DrpC identified homologs in apicomplexans and in representatives of the sister phylum Chromerida, but not in the representative of Perkinsozoa, Perkinsus marinus, or in any Ciliates representative. This suggests that *Tg*DrpC is restricted to apicomplexan and closely related organisms. Additionally, *Tg*DrpC was shown to be essential for parasite growth [34].

Here, we studied the putative fission mechanism in *T*. *gondii*. We showed that *Tg*DrpC has an essential function in mitochondria biogenesis, potentially via coordinating fission during parasite division. Moreover, we investigated the presence of the adaptor complex in *T*. *gondii*. Intriguingly, we found that *Tg*Fis1, a homolog of the human Fis1, an adaptor of Drp1, is not involved in mitochondrial fission in *T*. *gondii*. Indeed, *Tg*Fis1 depletion did not result in parasite death.

Our results highlight the essentiality of mitochondrial dynamics for the parasite and demonstrate that an unconventional, Apicomplexa-specific dynamin-related protein is at the core of this process.

## Materials and methods

### *T*. *gondii* parasite lines, maintenance and transfections

*T*. *gondii* tachyzoites were maintained in human foreskin fibroblasts (HFF) cultured in Dulbecco’s modified Eagle’s medium (DMEM) supplemented with 10% fetal bovine serum (FBS), 2 mM glutamine, and 25 μM gentamycin at 37°C and 5% CO_2_ in a humidified incubator. Transfections were carried out by electroporation using 10^^7^ freshly egressed or mechanically released parasites as previously described [35].

### Bioinformatics analysis

**Fig 1:**
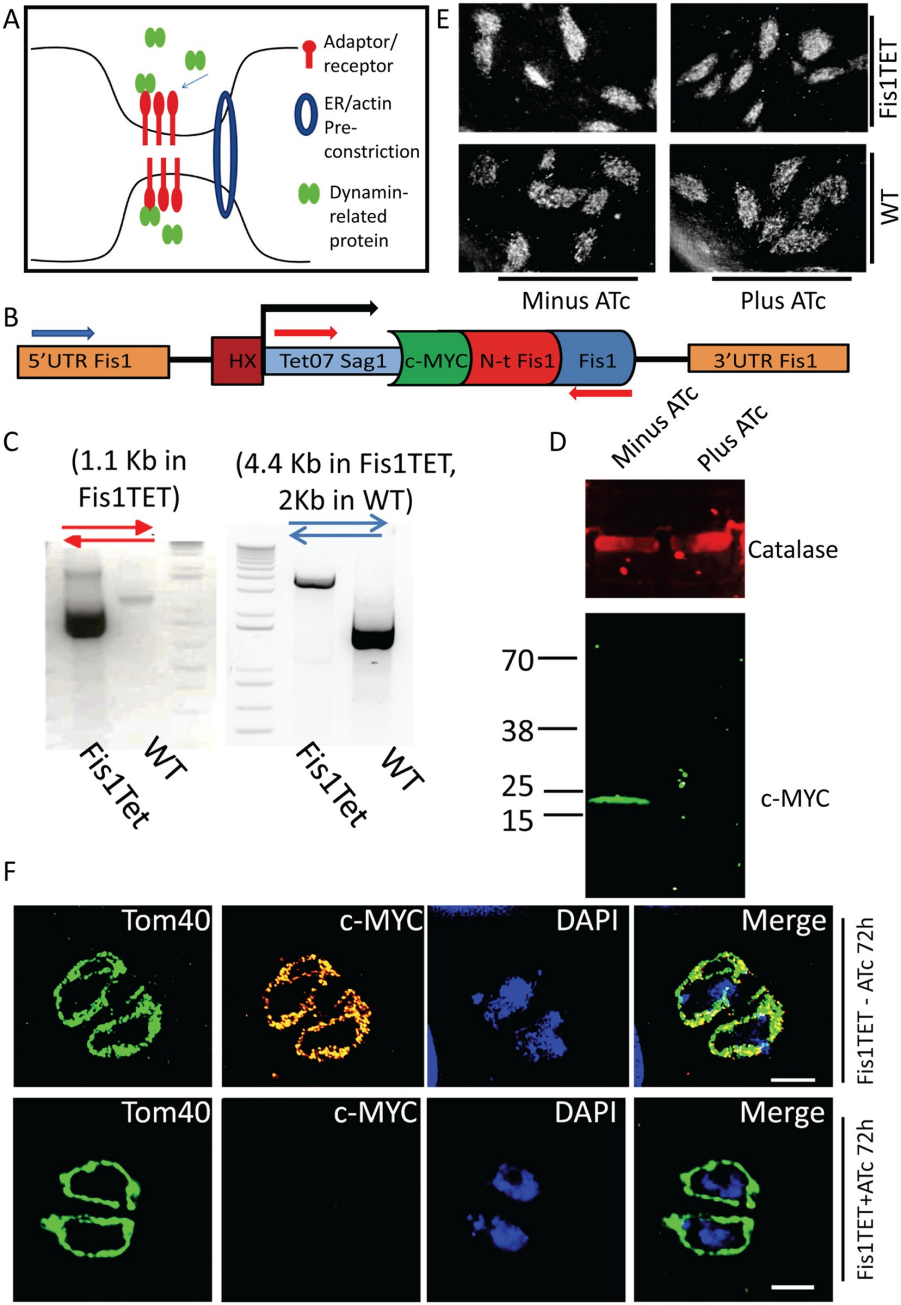
Conservation of the fission machinery in *T*. *gondii*: *Tg*Fis1 is not essential for mitochondrial fission A) Illustration of the main events that lead to mitochondrial fission in higher eukaryotes. ER and actin enwrap the mitochondrial membrane at future sites of fission, and an “adaptor complex” recruits Drp1/Dnm1, which forms spirals around the MOM and constricts it. (B) Scheme of the promoter replacement strategy adopted for the conditional KD of *Tg*Fis1; red and blue arrows indicate oligonucleotides designed to verify promoter exchange via genomic PCR. (C) Genomic PCR confirms the replacement of the endogenous promoter of *Tg*Fis1 with the inducible promoter Tet07-Sag1. A clonal parasite line was used for genomic PCR; primer positions and amplicon length are specified. (D) Confirmation of protein depletion upon addition of ATc for 48h in Fis1KD. In absence of ATc, western blot analysis shows the expected protein size of 17 kDa, tagged with c-MYC; the induction of *Tg*Fis1 knockdown with ATc shows that at 48 hours the protein is no longer detectable by western blot. (E) Plaque assay on parasites grown on HFF cells for 7 days in presence and absence of ATc shows that depletion of *Tg*Fis1 is not deleterious for parasite fitness. The images shown are representative of three experiments. (F) Immunofluorescence analysis shows that *Tg*Fis1 is evenly distributed in the mitochondrial outer membrane. In presence of ATc, the signal of Fis1 is no longer detectable, but mitochondria morphology is not affected. The experiment was performed in triplicate; representative images are shown. Scale bar: 2 μm.

Evidence for homology between fission proteins of human, yeast and plants (as reported in Fig 1B) and *T*. *gondii* was collected using reciprocal BLAST analysis. Sequences of Drp1 (Uniprot accession number: O00429), Dnm1 (P54861), Drp3 A/B (Q8S944 and Q8LFT2), Fis1 (Q9Y3D6), Mdv1 (P47025), Caf4 (P36130), Num1 (Q00402), Mdm36 (Q06820), Mff (Q9GZY8), Mid49/50 (Q96C03) and Elm1 (Q93YN4) were retrieved from UniProt database and BLASTP searches performed in ToxoDB. *Tg*DrpC shows high score when the BLAST is with human Drp1 (e-value of 2e^-06^), albeit lower than *Tg*DrpA and *Tg*DrpB. Reciprocal BLAST with *Tg*DrpC against NCBI identifies human Drp1 with high score (e-value of 6.5e^-6^). BLAST search with human Fis1 retrieves *Tg*Fis1 with an e-value of 1e^-12^.

BLAST searches using human Drp1 finds an additional putative gene (TGME49_326100) which seem to encode a fragment of a dynamin-like protein. However, consultation with the ToxoDB team suggested that this is likely a duplicate of the same sequence as that assembled into chromosome 1b (Omar Harb, personal communication).

### Homology modelling and molecular dynamics simulations

To construct a reliable homology for *Tg*DrpC, BLAST+ [2] was used to identify related sequences in the UniProt data base (The UniProt Consortium, 2016). The conserved N-terminal GTPase domain (300 residues) was then used to construct the homology model using SWISSMODEL [36, 37]. All potential models were evaluated manually with the model based on PDB:3L43 determined at a resolution of 2.3 Å, which shares a sequence identity of approximately 22%, being the most reliable (30% over the GTPase domain). To further assess the validity of the active site, GDP was included into the putative binding of the homology model and the GTPase-GDP complex model was optimized by energy minimization followed by a short molecular dynamics simulation using GROMACS [38]. GDP parameters were obtained in the Gromos54a7 forcefield from the ATP Repository (molID:35650) [39]. The simulation converged quickly resulting in an excellent binding pose for GDP consistent with its propose catalytic activity. Visual inspections, structural comparisons, least-squares superpositions and figures were prepared using Pymol [40].

### Plasmid construction

The plasmid *Tg*Fis1TET was generated as described in [41]. Fis1 5’UTR was amplified with primers Fis5’-fw and fis5’-rev, and the N-terminus of Fis1 was amplified with primers FisNterminus-fw and FisNterminus-rev. All fragments were cloned into the parental vector Tet07-Sag1-HX [41], which was transfected in the strain *TATIΔKu80ΔHX* and selected by HX selection [42]. Integration was confirmed using the primers FisTET integration fw 1 and FisTET integration rev, and FisTET integration fw 2 and FisTET integration rev.

To generate the plasmid *Tg*DrpC-YFP LIC, the C-terminal part of the gene was amplified using the primers DrpC-LIC fw and DrpC-LIC rev; the resulting PCR product was inserted into the plasmid pLIC-YFP by ligation independent cloning [43, 44]. Following transfection into the *Δku80* strain [43], transfected parasites were selected using pyrimethamine. Integration was confirmed using the primers DrpCYFP integration fw and DrpCYFP integration rev.

To generate the plasmids DD-GFP-DrpCwt and DD-GFP-DrpCtruncated, full length and truncated cDNA encoding *Tg*DrpC was amplified using oligonucleotides DD-DrpC_wt-_fw and DD-DrpC_wt-_rev, and DD-DrpCtruncated-fw and DD-DrpCtruncated-rev, respectively (Table 2). Subsequently, ligation through NsiI-NotI restriction into the vector p5RT70DDmycGFP-HX [32] was performed. The vector DD-DrpCwt was subsequently modified with Q5 modification kit (New England BioLabs, Catalog number E0554S) to delete the DD cassette, and obtain the plasmid GFP-DrpC_wt_, using primers Q5wt-fw and Q5wt-rev. Similarly, the vectors DD-GFP-DrpCDN and DD-GFP-DrpCGTPase only were obtained through modification of the DD-GFP-DrpC_wt_ plasmid, using the primers Q5_DN_-fw and Q5_DN_-rev, and Q5_GTPase only_-fw and Q5_GTPase only_–rev, respectively. Transfections were made in the RH parental strain.

**Table 2:** Oligonucleotides used.

Oligo name	Sequence
Fis5’fw	gggTCATGAGTCTCTTTTGAAGACGTGCACCG
Fis5’rev	ggGGATCCTCTCGTACAGTGCTCACAAAAAACG
FisNterminusfw	cccAGATCTATGGAAGACTCCAACTTCAGTC
FisNterminusrev	gggACTAGTGGGCGAAACACGCAAGTAAC
FisTET integration fw 1	GCTGCACCACTTCATTATTTCTTCTGG
FisTET integration fw 2	TCATGAGTCTCTTTTGAAGACGTGCACCG
FisTET integration rev	GCTATAAACACAGCCGAGGCGAC
DrpC-LIC fw	TACTTCCAATCCAATTTAATGCCCGAG
DrpC-LIC rev	TCCTCCACTTCCAATTTTAGCAGCC
DrpCYFP integration fw	GCGCCACTCACGACGAAG
DrpCYFP integration rev	ATGGGCACCACCCCGG
DrpCYFP integration fw 2	TTTGTGATGCTCGTCAGG
DrpCYFP integration rev 2	CTGGAACCCTTCCATACTG
DD-DrpC_wt_	CCATGCATCGAACGCGCTGCCGCGTC
DD-DrpC_w_	CCTTAATTAATTACGCCCCATTCAACGGTGACGGAAGC
Q5wt-fw	TTTAGATCTAAAAGGGAATTCAAGAAAAAATG
Q5wt-rev	GCCATGGCCATGCATAGT
Q5_DN_-fw	GAGCATGGGCgcgACGACCCTTCTC
Q5_DN_-rev	TGCTGCCCGAAGACGACA
Q5_GTPase only_-fw	TAATCACCGTTGTGCTCAC
Q5_GTPase only_–rev	CTCACTCAAGAGGCTCTG
DD-DrpCtruncated-fw	CCTTAATTAATTACGCCCCATTCAACGGTGACGGAAGC
DD-DrpCtruncated-rev	CCATGCATGGAGCCTTCGAGAGTTCATTCTCTCTGCACCTCC

### Immunoblot analysis

Parasites were cultivated for the indicated times in the following conditions: the line *Fis1TET* was grown in presence or absence of 0.5 μg/mL ATc for 72 hours; the strain *TgDrpC-YFP* was grown in normal medium; parasites *TgDrpC-U1* were grown for 24, 48 and 72 hours in presence of 50 nM of Rapamycin; the line *DD-GFP-DrpCDN* was grown with 0.5 μM of Shield-1 for 0, 4, 8 or 24 hours; the strains *DD-GFP-DrpCwt*, *DD-GFP-DrpCGTPase only* and *DD-GFP-DrpCwt* were cultivated in presence or absence of 1 μM Shield-1 for 24 hours. Parasites were subsequently harvested and western blot analysis of total parasite lysates were performed as described previously [45], using the antibodies indicated in the figures and figure legends.

### Plaque assay

For plaque assays, the clonal lines *Fis1TET* and *DD-GFP-DrpCDN* were grown for 7 days in the presence or absence of 0.5 μg/mL ATc or 1 μM of Shield-1, respectively. Fixation, staining and visualization of plaques were performed as previously described [32].

### Immunofluorescence analysis

Immunofluorescence analysis was carried out on cover slips. Cells were fixed with 4% paraformaldehyde for 20 minutes, then a permeabilisation step using 0.2% Triton X-100 in PBS was performed for 20 minutes, followed by a blocking step using 2% bovine serum albumin in PBS for 20 minutes, then stained for 60 minutes with the indicated antibodies. Primary antibodies used were: mouse anti c-MYC (Sigma, M-4439), rabbit anti-catalase, rabbit anti-Mic4 and rabbit anti-GAP45 (gifts from Dominique Soldati), mouse anti-HSP60 (gifts from Boris Striepen), rabbit anti-Tom40 (gifts from Giel Van Dooren), rat anti-DrpB, rabbit anti-Rop13 (gift from Peter Bradley). Subsequently, secondary stain was performed for 60 minutes. Secondary antibodies used were goat anti-mouse, goat anti-rabbit or goat anti-rat AlexaFluor 350, AlexaFluor 488, AlexaFluor 594 or AlexaFluor 633-conjugated antibodies (Life Technologies). These reactions were carried out at room temperature. Co-localisation analysis was performed using FIJI plugin “JACoP” to determine Manders’ coefficient, counting at least 20 images per condition [46].

### Microscopy

Widefield images were acquired in z-stacks of 2 μm increments and were collected using an Olympus UPLSAPO 100x oil (1.40NA) objective on a Deltavision Core microscope (Applied Precision, GE) attached to a CoolSNAP HQ2 CCD camera. Deconvolution was performed using SoftWoRx Suite 2.0 (Applied Precision, GE). Video microscopy was conducted with the DeltaVision Core microscope as above. Normal growth conditions were maintained throughout the experiment (37°C; 5% CO_2_). Further image processing was performed using ImageJ64 software.

Moreover, an ELYRA PS.1 microscope (Zeiss) was used for super-resolution microscopy (SR-SIM), as described in [47].

## Results

### *T*. *gondii* does not possess a conserved mitochondrial fission complex

To identify candidates involved in *T*. *gondii* mitochondrial fission, we performed bioinformatics analysis to assess the conservation of known fission factors from yeast, human and plants in the genome of the parasite (illustrated in Fig 1A). We could only identify putative homologs for the dynamin component (*Tg*DrpA, *Tg*DrpB and *Tg*DrpC) and for the fission component (*Tg*Fis1) (Fig 1B). We ruled out *Tg*DrpA and *Tg*DrpB, since earlier analyses indicated no major involvement of these dynamin-related proteins in mitochondria function. Therefore, we hypothesised that *Tg*Fis1 (TGME49_263323) and *Tg*DrpC (TGME49_270690) could be involved in mitochondrial fission in *T*. *gondii*, and set out to examine these two proteins in detail.

### Conditional knock-down of Fis1 does not impair mitochondria biogenesis

The fission-protein 1 (*Tg*Fis1)—a tetratricopeptide repeat domain-containing protein that is essential for mitochondrial fission in yeast—is highly conserved in *T*. *gondii*, as shown by Padgett et al. [48], who identified it as a tail-anchored protein, and showed that when ectopically expressed it localises to the mitochondrion.

To investigate the role of *Tg*Fis1 in *T*. *gondii*, we employed the tetracyclin inducible transactivator system [49] to generate a knockdown parasite line for *TgFis1*. Briefly, the endogenous promoter of *TgFis1* was replaced via homologous recombination, as previously described [41, 42], resulting in a parasite line where *Tg*Fis1 is N-terminally tagged with c-MYC epitope tag and is under the control of the conditional promoter T7Sag1 (Fig 1C) [49]. Promoter replacement was confirmed by PCR and the regulation is shown by western blot analysis (Fig 1D and 1E). As shown in Fig 1G, in absence of ATc the *Tg*Fis1-Myc signal overlaps with the signal of the mitochondrial marker *Tg*TOM40 [50]. Immunoblot and immunofluorescence analysis demonstrate that the signal of *Tg*Fis1 is undetectable at 72 hours post induction with ATc. Plaque assay measuring parasite growth for 7days shows that depletion of *Tg*Fis1 does not have a deleterious effect on parasite proliferation (Fig 1F); furthermore, no defect in mitochondria morphogenesis is observable by immunofluorescence analysis (IFA) (Fig 1G). These results are confirmed by a CRISPR/Cas9 genome-wide screen, where *Tg*Fis1 is classified as “dispensable”, with a phenotypic score of +0.94 [51]. We conclude that *Tg*Fis1 is not required for the growth of *T*. *gondii* tachyzoites in cell culture, and that it does not play a central role in the control of mitochondrial morphology under normal growth conditions.

### *Tg*DrpC is an apicomplexan dynamin-related protein and *in silico* studies predict its GTPase domain to be active

To understand *Tg*DrpC function in *T*. *gondii*, we first performed a detailed *in silico* analysis. *Tg*DrpC is a highly divergent, potential dynamin-related protein, characterised by a GTPase domain at its N-terminus, and by a tail domain which is largely disordered. While the first 118 residues of *Tg*DrpC, as well as the C-terminal tail, share no detectable sequence similarity with any known structures, the putative GTP binding domain shows low yet marked sequence identities with a range of other GTP binding proteins.

To provide molecular insight into the putative GTPase activity, a homology model was constructed using SWISSmodel [36]: the closest model in the protein database as identified by BLAST [52] is the crystal structure of the human dynamin 3 GTPase domain (PDB: 3L43), determined by the Toronto Structural Genomics Consortium at a resolution of 2.3 Å. The GTPase domain of human dynamin 1 and dynamin 3 are highly conserved, with a sequence similarity of over 80% for the GTPase domain (identity 56%) and more importantly, all GTP contacting residues are fully conserved. As *Tg*DrpC and human dynamin 3 share a sequence identity of 31% over the GTPase domain (amino-acids 118–401), a reliable homology model for the overall structure of this domain can be calculated for *Tg*DrpC; however, as shown in S1A Fig, there are a number of insertions and deletions, in particular in the C-terminal side of *Tg*DrpC GTPase domain. We predict that while these differences will lead to significant local structural changes, the overall fold will be conserved. To further validate GDP/GTP binding, GDP was included in the putative binding pocket and the structure of the resulting GTPase-GDP complex was optimized by molecular dynamics simulations.

**Fig 2:**
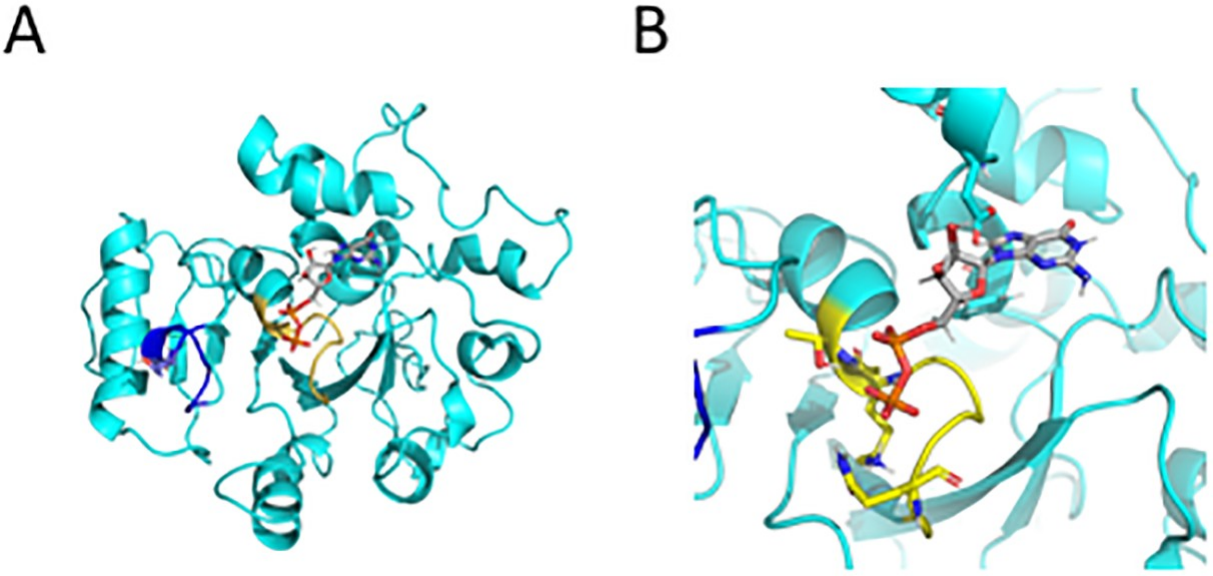
Modelling of the GTP binding domain of *Tg*DrpC (A) Monomer model for *Tg*DrpC GTPase domain. The P-loop responsible for tri/diphosphate binding is indicated in yellow, while the switch I region with the putative MG^2+^ coordinating threonine is in blue. (B) close-up of the GDP binding site after molecular dynamics simulation with key residue interacting to the co-substrate shown in ball-and-stick representation, yellow for residues in the P-loop, cyan for residues interacting with the ribose and the guanine.

As shown in Fig 2A and 2B, the GTP binding site is mainly conserved, with the diphosphate-contacting P-loop (shown in yellow) displaying only small changes in sequence with its characteristic GX_4_GKT motif and structure. In this region, the residues Ser41 and Lys44 (numbering corresponding to the human dynamin 3), which form a salt bridge to the β-phosphate group, are conserved, while the following Ser44 and Ser45 residues–which in human dynamin 3 form H-bonds to the β-phosphate and α-phosphate, respectively—correspond to threonine residues able to form the same interactions in *Tg*DrpC. Importantly, the switch I region shown in blue, contains the conserved threonine residues that may serve in the GTP hydrolysis by interacting with the Mg^2+^ and the γ-phosphate [53].

In addition, the *Tg*DrpC equivalent to the Switch II region, which in other GTPases contains either a conserved glutamine or histidine residue that can activate the water molecule for GTP hydrolysis, harbours a lysine residue close-by, which could fulfil the same function. In contrast, the guanine-contacting residues (Lys206, Asp208, Asp211 and Asn236 in the human dynamin 3 GTPase structure, shown in blue in S1A Fig) are not conserved in *Tg*DrpC, and as a result, some reorganisation of the loop regions were required in the molecular dynamics simulation to accommodate GDP/GTP binding. The 3D homology model, shown in Fig 2b, clearly supports the notion of GTPase activity; however, no further conclusion about which residue acts as water-activating residue to perform the nucleophilic attack should be drawn, as in this part of the model the sequence similarity with human dynamin 3 is significantly lower.

Based on the homodimeric human dynamin 3 structure [37], the *Tg*DrpC homology model adopts a similar homodimer (Fig 2C), though the oligomeric structure of the full-length *Tg*DrpC remains to be elucidated. Finally, the C-terminus of the protein is highly disordered. Clustal-Omega alignment of *Tg*DrpC homologs in Apicomplexa (Table 3, S1B Fig) shows that there are two highly conserved regions in the tail (highlighted in red). These regions, however, do not correspond to any canonical GTPase effector domain (GED) or middle domain, which are commonly found in dynamin-related proteins.

**Table 3:** accession numbers of sequences used for alignment.

Sequence	UniProt accession number
*Tg* DrpC	S8GIW4_TOXGM
*Ta* DrpC	Q4UHJ0_THEAN
*Bb* DrpC	A7AS48_BABBO
*Pf* DrpC	Q8I5M3_PLAF7
*Pv* DrpC	A5JZZ2_PLAVS
*Pb* DrpC	A0A077XK90_PLABA
*Cp* DrpC	Q5CW16_CRYPI

### *Tg*DrpC forms foci at the mitochondrial periphery and basal end

**Fig 3:**
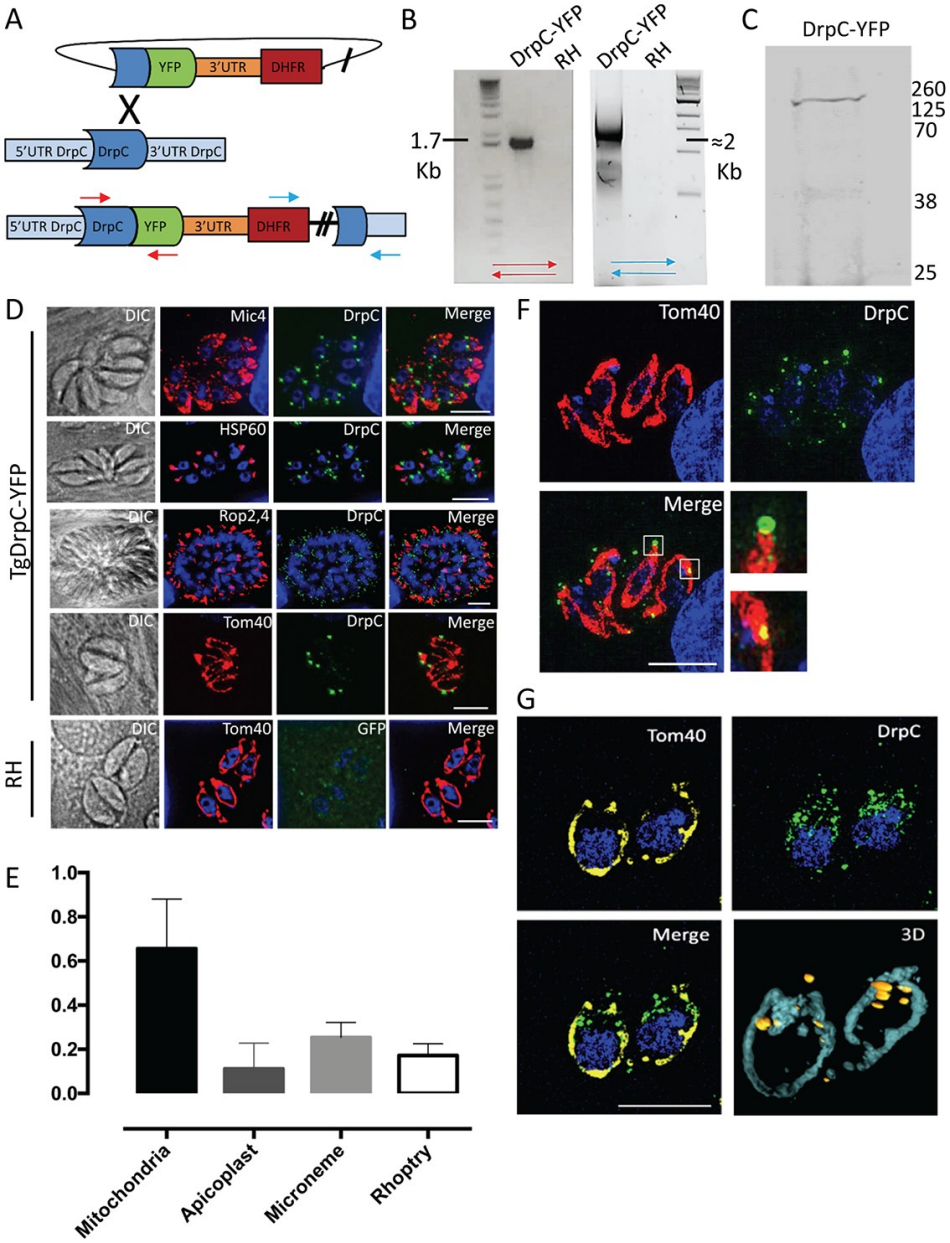
Endogenous tagging of *Tg*DrpC reveals a mitochondria localisation. (A) Strategy for the endogenous tagging of *Tg*DrpC with YFP in RHΔku80 parasite strain: The ligation-independent cloning vector LIC-YFP was integrated at the 3’ end of *Tg*DrpC, through a single-crossover mechanism. (B) PCR analyses using the primers indicated in (A) confirms the integration of the tagging construct at 5’ and 3’ ends. (C) Western blot analysis using α-GFP antibody on the *Tg*DrpC-YFP clonal line verifies the expected protein size of ≈160 kDa. (D) Immunofluorescence images showing the co-localisation between *Tg*DrpC and the indicated organelles (Mic4, micronemes; HSP60, apicoplast; Rop2-4, rhoptries; Tom40, mitochondrion). (E) Quantification of colocalisation between *Tg*DrpC and the indicated organelles; the Manders’ coefficient (average of n>20 values) is reported in the y axis. (F) *Tg*DrpC puncta (green) have different shapes and sizes, varying from spirals to smaller dots on the membrane; 3-D reconstruction (G) confirms that the vast majority of *Tg*DrpC aggregates are in close proximity with mitochondria. Scale bar: 5 μm.

To assess the localization of *Tg*DrpC, we generated a knock-in strain where *Tg*DrpC is endogenously tagged at its C-terminus with YFP, as confirmed by PCR (Fig 3A and 3B). Western blot analysis performed with the obtained clonal line *TgDrpC-YFP* confirms the expression of the fusion protein, which has the predicted mass of ≈160 kDa (Fig 3C).

Dynamin-related proteins studied in other systems form a diffuse cytoplasmic pool until recruited to the target membrane, where they oligomerize and form spirals [13, 54]. Similarly, our immunofluorescence analysis of *Tg*DrpC-YFP detected a faint but specific cytoplasmic signal, alongside a distinct pattern of punctate structures that appear associated with the mitochondrial membrane (Fig 3D and 3E). The number and size of these structures are highly variable: when viewed from above, it is possible to visualise spiral-like structures of *Tg*DrpC around the mitochondrial tubule (Fig 3F, upper inset), while in other cases the puncta are small and form dots (Fig 3F, lower inset). Evaluation of images from 3D reconstruction confirm that most puncta are in close proximity to the mitochondrion (Fig 3G). Quantification indicates that the mitochondrion is the only organelle that shows a strong co-localisation with *Tg*DrpC (Manders’ coefficient ≈0.7); in particular, while the foci at the periphery are not always in contact with the organelle, the ones at the basal end of the parasite consistently co-localise with the mitochondrion (Fig 3E).

### *Tg*DrpC is recruited to the basal end of mitochondria during endodyogeny

**Fig 4:**
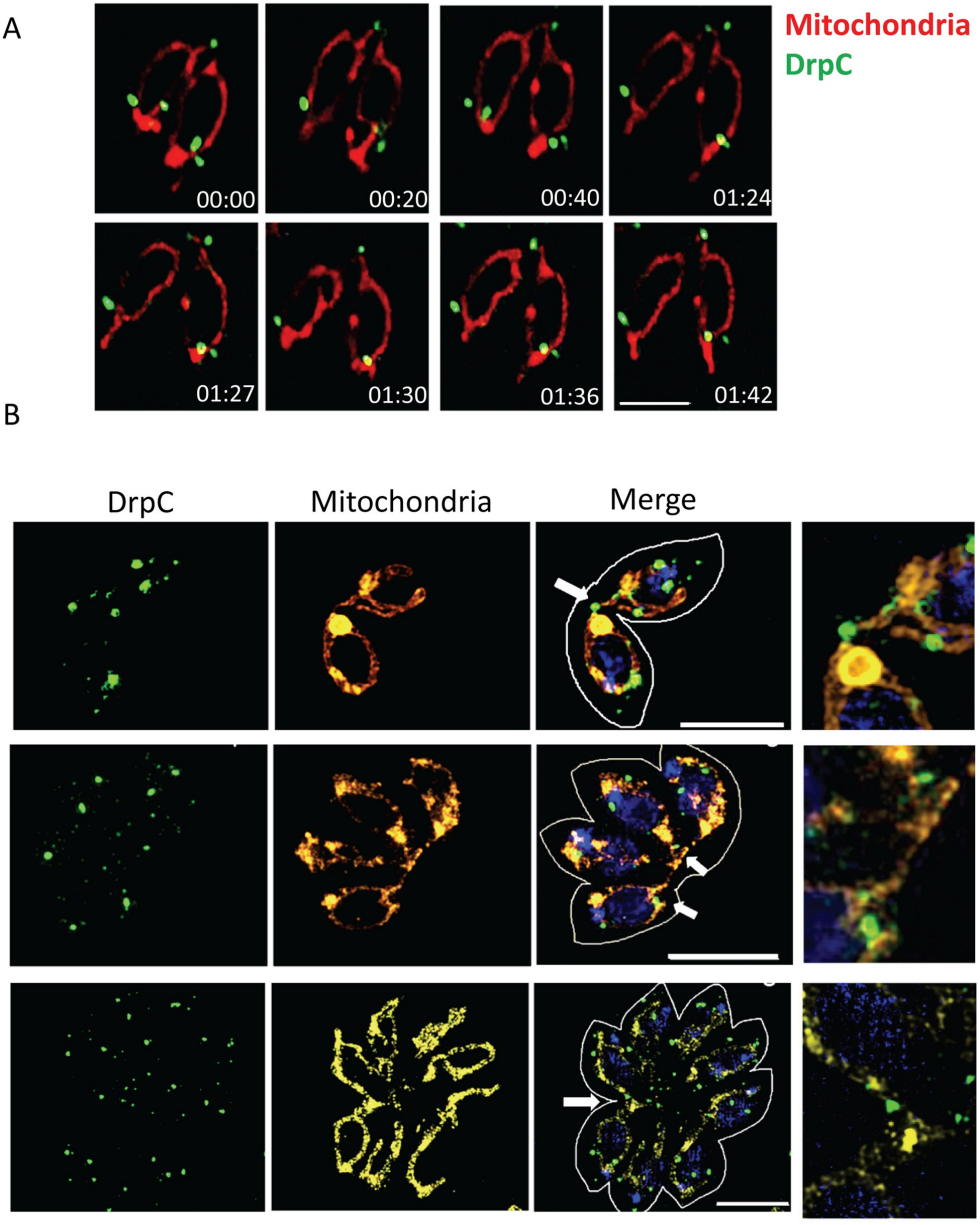
Recruitment of *Tg*DrpC at interconnected mitochondria during replication. (A) Time-lapse analysis of parasites *Tg*DrpC-YFP/TGME49_215430-tdTomato with interconnected mitochondria (in red, TGME49_215430-tdTomato; in green, *Tg*DrpC-YFP). *Tg*DrpC is at the mitochondria periphery and at the basal interconnection; while the puncta at the periphery are more static, the signal at the basal end is highly dynamic. Time is indicated in minutes. Scale bar: 5 μm. (B) SIM microscopy shows that the puncta at the basal end coincide with sites of constriction of the MOM (white arrows). Scale bar: 5 μm.

In the last stages of endodyogeny, newly-formed mitochondria migrate into the two developing daughter cells [16]; they remain attached for an unknown period at the basal part, where they are eventually separated. To follow the localization of *Tg*DrpC during endodyogeny, we performed live cell imaging on parasites expressing the outer mitochondrial marker TGME49_215430 fused to tdTomato [5] in the strain *TgDrpC-YFP*. We focused our analysis on parasites at the late stages of replication, when mitochondria are inside the newly formed daughter, but still connected at the basal end (S1 Video). As shown in Fig 4A, the puncta of *Tg*DrpC are both at the periphery and at the basal connection between the two mitochondria. While the *Tg*DrpC puncta found at the periphery of the mitochondria remain static, the signal at the basal connection of the mitochondria is highly dynamic. In the example shown in Fig 4A, at time point 00:20 two distinct *Tg*DrpC foci are at the basal interconnection; at 00:40, a constriction is noticeable in one of the foci, and at 1:27 *Tg*DrpC seems to be at the terminal end of the mitochondria tubule, and the connection is no longer detectable. Interestingly, one of the *Tg*DrpC foci persisted at the basal end of the mitochondria, even twenty minutes after the cleavage step occurred. These behaviours are in support of a *Tg*DrpC role in mitochondrial fission.

To provide further support for this model, we performed structured illumination microscopy (SIM) on the *TgDrpC-YFP/TGME49_215430-tdTomato* strain, choosing vacuoles containing two, four or eight parasites which show mitochondria that are still interconnected. Multiple puncta of *Tg*DrpC are visible at the interconnections of the mitochondria at the basal end (Fig 4B). Importantly, in some cases an invagination of the mitochondrial tubule is seen and what seems like constriction of the membrane colocalises with *Tg*DrpC signal.

Together, our results from live and fixed samples indicate that *Tg*DrpC is present at the basal end of mitochondria during the last stages of endodyogeny, and that *Tg*DrpC may constrict the interconnected mitochondria, mirroring the behaviour of Drp1 and Dnm1 in humans and yeast [55].

### Conditional knock-down of *Tg*DrpC results in interconnected mitochondria

**Fig 5:**
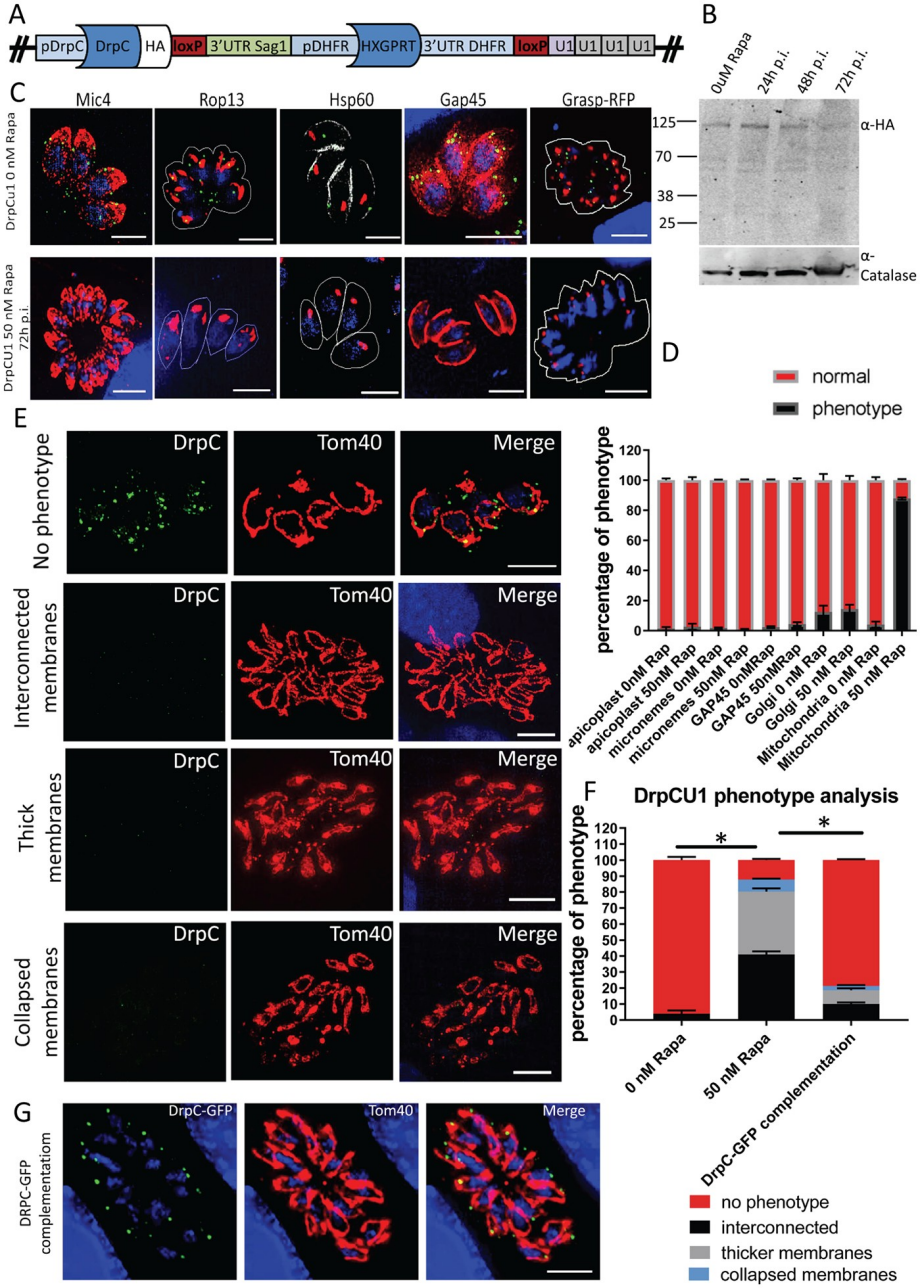
Inducible Knock-down of DrpC has a specific mitochondrial effect. (A) Scheme of the genomic locus after integration of the DrpC-U1 construct, as detailed in Pieperhoff et al., 2015. (B) Western Blot analysis showing efficient downregulation of *Tg*DrpC 72 hours post Rapamycin induction. The membrane was probed with α-HA and α-Catalase antibodies. (C) Immunofluorescence analysis shows that *Tg*DrpC signal is no longer observable at 72 hours post induction; at this time, the distribution of micronemes and rhoptries, probed with Mic4 and Rop13, was not affected in Rapamycin-treated parasites; similarly, the morphology of apicoplast (αHSP60), IMC (αGAP45) and Golgi (GRASP-RFP) appeared normal, as quantified in (D). (D) Organelle phenotypes were scored in the following way: for micronemes and rhoptries, an apical distribution was counted as “normal”; for the apicoplast, both location (at the apical part of resting parasites) and number (one per parasite in a vacuole) were assessed; for the Golgi, correct distribution above the nucleus and morphology (i.e., not fragmented) were checked; the IMC stain forming the “banana shape” typical of tachyzoites was scored as normal. For each data set, 100 parasitophorous vacuoles were counted. This was performed in triplicate. In contrast, as shown by Immunofluorescence analysis in (E) and quantified in (F), induced parasites showed severe morphology defects in the mitochondrion, classified as “interconnected mitochondria” (41%), “thick mitochondrial membranes” (39.2%) and “collapsed mitochondria” (7%) Scale bar: 5μm. (G) Complementation experiments through transient transfection of plasmid p5RT70-GFP-DrpC in Rapamycin-treated parasites led to rescue of the mitochondrial phenotype, as quantified in (F). Quantification in (F) was obtained through comparison of three independent experiments, each with at least 300 vacuoles. Error bars show SD. Asterisks indicate significant difference (P<0.001 multiple t-test).

We reasoned that if involved in fission, *Tg*DrpC depletion would inhibit mitochondrial separation after division [13, 54]. We analysed the phenotypic consequences of *Tg*DrpC depletion using the U1 system [34] to obtain a *TgDrpC-U1* inducible mutant, where *Tg*DrpC is tagged with an HA epitope tag, and addition of rapamycin results in *Tg*DrpC mRNA degradation (Fig 5A). In accordance to our previous findings (also shown here, S2A Fig), we observed that *Tg*DrpC is essential for parasite survival.

Western blot analysis confirmed that at 72 hours post induction *Tg*DrpC-HA is highly reduced at the protein level (Fig 5B). Similarly, we observe no puncta of *Tg*DrpC by IFA at 72 hours post-induction (Fig 5C, lower panel). In agreement with *Tg*DrpC involvement in mitochondrial biogenesis, at this time point 90% of mitochondria show abnormal morphology, while in non-induced *Tg*DrpC-HA-U1 96% of the mitochondria show the typical lasso-shape morphology (Fig 5D and 5E). We identified three different mitochondrial phenotypes in the induced parasites: 41% of the vacuoles contain mitochondria that remain interconnected with each other; 39.2% of the vacuoles show mitochondria with what seem to be swollen membranes, a phenotype we refer to as “thick membranes”; finally, ~7% of the vacuoles show small mitochondria shaped like closed circles (Fig 5E and 5F). In contrast, at the same time point we found that *Tg*DrpC down-regulation has no significant effect on other organelles such as the apicomplexan-specific secretory organelles (micronemes and rhoptries) and apicoplast; likewise, rosette organisation is not significantly affected by *Tg*DrpC knock-down, and Golgi morphology, as assessed with the signal of transiently expressed GRASP-RFP marker [56], shows no significant differences in induced and non-induced parasites 72 hours after induction (Fig 5C and 5D). When *Tg*DrpC knock-down was analysed at later time points, a drastic morphological change was observed, where mitochondria completely lost their shape. At this late time point some parasites also showed additional defects, such as disruption of the IMC, which most likely represents a non-specific secondary effect caused by depletion of *Tg*DrpC (S2B Fig).

Finally, to correlate the observed mitochondria phenotype to *Tg*DrpC depletion, we carried out a complementation experiment by transiently expressing a second copy of *Tg*DrpC tagged with GFP (GFP-DrpC). GFP-DrpC localises to the MOM in rapamycin treated and non-treated parasites, and fully rescues the mitochondrial morphological defect when expressed in induced *TgDrpC-U1* parasites (Fig 5F and 5G). Taken together, these data support a role for *Tg*DrpC in mitochondria morphogenesis.

### The expression of a dominant-negative form of DrpC impairs correct mitochondria segregation

The variable morphological phenotypes observed upon prolonged *Tg*DrpC depletion (S2 Fig) raise the possibility that this protein could contribute to additional functions during parasite replication; an alternative explanation is that some of the morphologies represent a secondary outcome occurring in response to DrpC depletion. We reasoned that a rapid induction of protein depletion would allow us to identify the primary function of *Tg*DrpC.

To achieve this, we employed the ddFKBP-system [57] to rapidly express a dominant-negative version of *Tg*DrpC. This strategy is based on previous research showing that expression of dynamins with mutations in the GTPase domain has a dominant-negative effect. In these mutants, loss of the GTPase activity impairs the dynamin function, but does not affect membrane recruitment. As a result, competition between the endogenous and the ectopic mutant over membrane binding leads to functional impairment of endogenous *Tg*DrpC [17, 58, 59].

**Fig 6:**
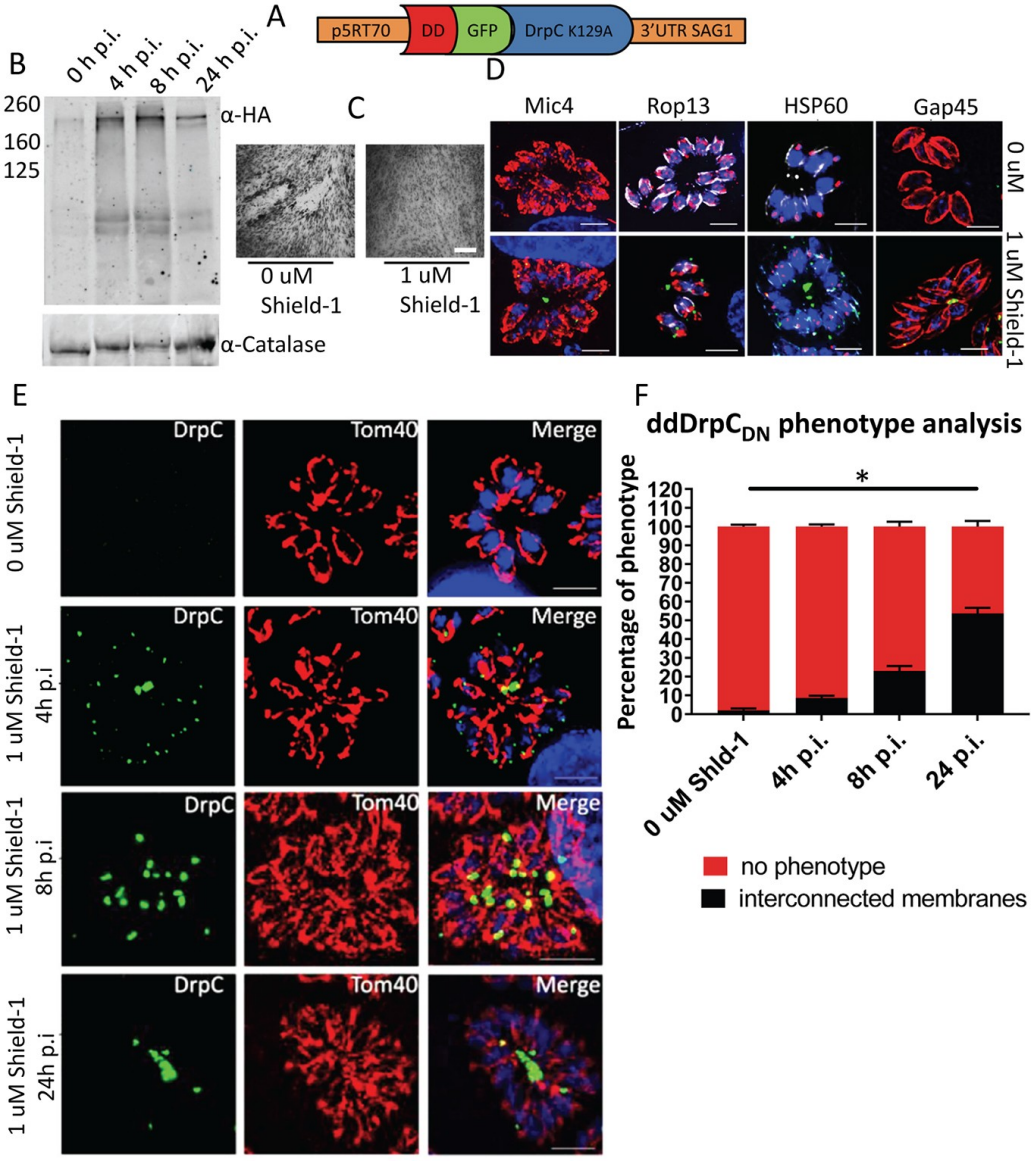
Over-expression of a dominant-negative form of *Tg*DrpC leads to interconnected mitochondria. A) Scheme of the plasmid DD-GFP-DrpCDN. (B) Western blot analysis with the indicated antibodies shows that Shield-1 induction efficiently regulates the expression of DD-GFP-DrpCDN as soon as 4 hours post induction. (C) Plaque assay shows that induction of DD-GFP-DrpCDN causes parasite death. Parasites were grown for 7 days on HFF cells in presence and absence of 1μM Shield-1. The experiment was performed in triplicate; representative images are shown. (D) The effects of DD-GFP-DrpCDN expression were assessed 24 hours after Shield-1 induction; immunofluorescence analysis of the specific markers Mic4, Rop13, HSP60 and Gap45 shows no effect on secretory organelles, apicoplast or IMC morphology. (E and F) Eight hours after stabilisation, DD-GFP-DrpCDN shows an accumulation at the basal part of the mitochondria, which increasingly appear interconnected (23% of the total); 24 hours after induction, more than half of the parasites show abnormally interconnected mitochondria, and DD-GFP-DrpCDN accumulates at the interconnections (scale bar: 5 μm). At least 100 parasitophorous vacuoles were counted in triplicate. Error bars represent SD from the three independent experiments. Asterisks indicate significant difference (P<0.001 multiple t-test).

We generated a line (*DD-GFP-DrpCDN*) whereby ddFKBP-DrpC(K129A) is randomly integrated in the genome of RH parasites; efficient regulation was demonstrated by western blot analysis following 0 to 24 hours induction with 0.5 μM Shield-1 (Fig 6B). We detected very low levels of DD-GFP-DrpCDN in the absence of Shield-1, and 4 hours after induction, full expression levels are reached, with no significant change after 8–24 hours.

In agreement with our observations for *Tg*DrpC depletion, plaque assays showed impaired growth in response to Shield-1 induced expression of dominant-negative *Tg*DrpC (Fig 6C). Likewise, immunofluorescence analysis of *DD-GFP-DrpCDN* parasites grown in presence of Shield-1 for 24 hours shows that, while no effect is observed on the biogenesis of the secretory organelles, the apicoplast and the IMC (Fig 6D), a mitochondrial defect is evident, as more than half of the vacuoles show an “interconnected mitochondria” phenotype (Fig 6E and 6F). Interestingly, DD-GFP-DrpCDN is both at the periphery and at the basal part of the mitochondrion at 4 hours post-induction, when only 9% of the vacuoles show interconnected mitochondria. At 8 hours post-induction, almost all the signal is accumulated at the basal part of the mitochondrion, and the proportion of interconnected mitochondria rises to 23%; at 24 hours post-induction, 53.6% of the vacuoles have this phenotype, and show an accumulation of DD-GFP-DrpCDN signal at the interconnection regions. Importantly, at these time points neither of the other mitochondrial phenotypes seen upon U1-knockdown of *Tg*DrpC (thicker membrane or closed-circled mitochondria) was observed. These data support the function of *Tg*DrpC in mitochondrial fission and suggest that the additional phenotypes observed upon extended knockdown may result from downstream effects.

Notably, the over-expression of the wild-type copy of *Tg*DrpC (*DD-GFP-DrpCwt*) does not influence parasite fitness; Shield-1 mediated stabilisation of DD-GFP-DrpCwt causes the protein to form puncta at the mitochondrion, and no mitochondrial phenotype is observed upon induction (S3C Fig). The stronger fluorescence signal obtained by overexpression of DD-GFP-DrpCwt allowed us to follow *Tg*DrpC localisation over longer times in using time lapse analysis and confirmed the data obtained for endogenous tagged *Tg*DrpC, supporting the proposed role of *Tg*DrpC in mitochondrial fission (S2D and S2E Fig).

**Fig 7:**
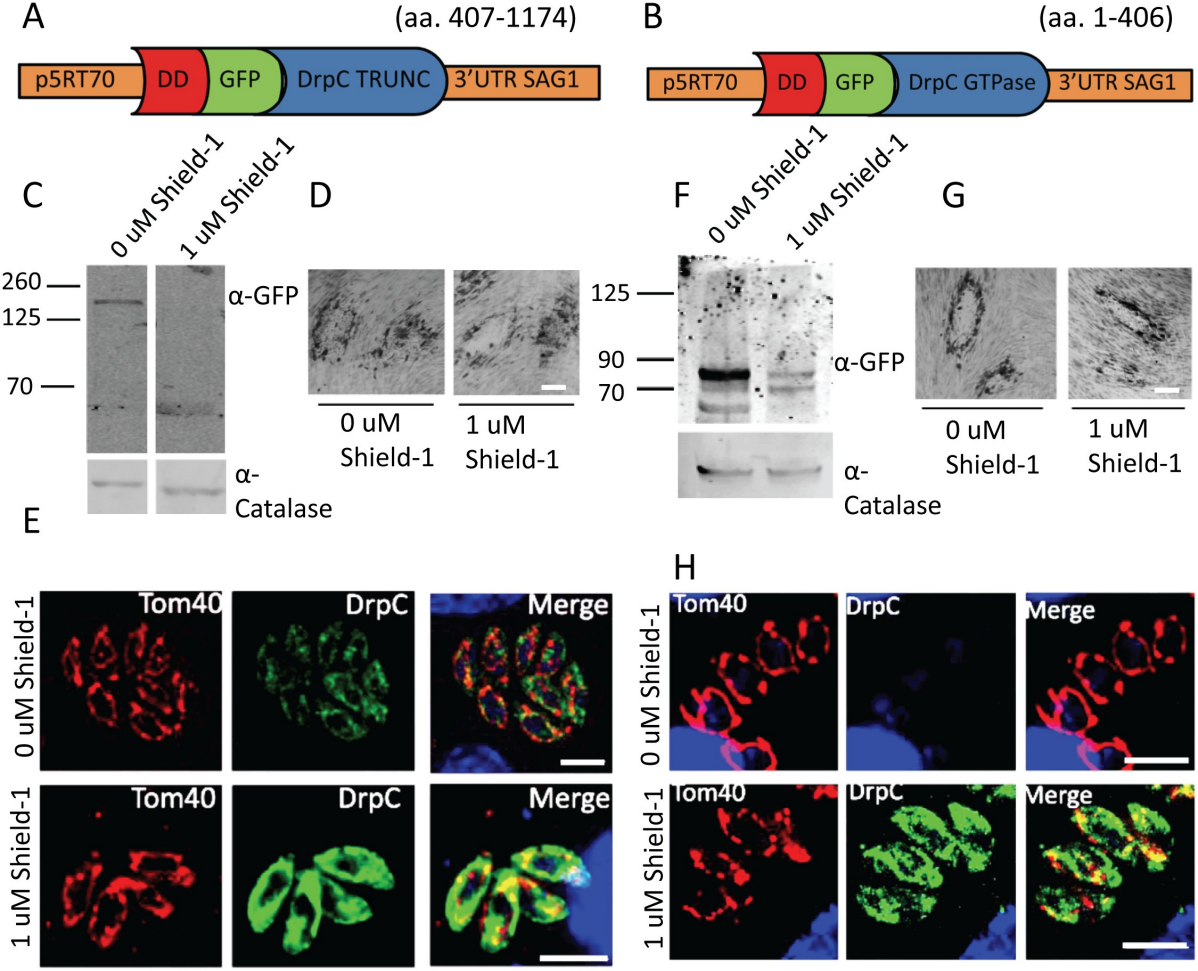
Truncated *Tg*DrpC does not localise to the mitochondrion. (A) and (B) Schematics of the plasmids DD-GFP-DrpCtruncated (amino-acids 407–1174) and DD-GFP-DrpCGTPase only (amino-acids 1–406). (C) Western blot analysis shows efficient DD-GFP-DrpCtruncated stabilisation 24 hours after Shield-1 induction. (D) Plaque assay analysis shows that overexpression of DD-GFP-DrpCtruncated does not impair parasite fitness. Parasites were grown for 7 days on HFF cells in presence and absence of 1μM Shield-1. (E) Immunofluorescence analysis shows that DD-GFP-DrpCtruncated localises to the cytoplasm and does not form puncta. (F) Immunoblot analysis of clonal DD-GFP-DrpCGTPase only parasites in presence and absence of Shield-1 using the indicated antibodies. (G) DD-GFP-DrpCGTPase only parasites grown in presence of 1 μM of Shield-1 for 7 days do not show any growth defect compared to non-induced controls. (H) Immunofluorescence analysis shows that DD-GFP-DrpCGTPase only localise to the cytoplasm. Scale bar: 5 μm.

Since our data provides support for *Tg*DrpC involvement in mitochondrial morphogenesis, we were interested to better understand its potential interaction with the mitochondrion. To gain some information about the minimal mitochondrial targeting domain of *Tg*DrpC, we obtained the parasite strains *DD-GFP-DrpCtruncated* and *DD-GFP-DrpCGtpase only*, which express only the GTPase or the C-terminus domain of *Tg*DrpC, respectively (Fig 7A and 7B); western blot analysis confirmed inducible expression of the truncated proteins (Fig 7C and 7F). We found that the over-expression of DD-GFP-DrpCtruncated and of DD-GFP-DrpCGtpase only does not impair parasite fitness (Fig 7D and 7G); these truncated versions of *Tg*DrpC do not associate with the mitochondrion, but localise diffusely in the cytosol of the parasite (Fig 7E and 7H). Moreover, our attempts to complement *Tg*DrpC knockdown with these truncated forms of the protein were unsuccessful. Since a dominant negative version of *Tg*DrpC localises correctly to the mitochondrial periphery, we conclude that the full-length protein is required for association with the mitochondria, though no GTPase activity is required.

## Discussion

Mitochondrial fission is crucial for the control of organelle morphology and function. Here, we investigated the role in *T*. *gondii* of conserved components of the fission machinery.

We show for the first time that a dynamin-related protein, termed *Tg*DrpC, is essential for mitochondrial biogenesis; we propose that it plays a role in mitochondrial fission at the end of cytokinesis. *Tg*DrpC localises in puncta, and its recruitment at the basal end of the mitochondrion is evident in the last steps of endodyogeny. It has been suggested that, after the entry of the new mitochondria into the budding daughter cells, the mitochondrial connection between the two branches is cleaved [16]. We show by time-lapse and fixed microscopy that *Tg*DrpC is at the interconnections until they are no longer visible. In addition to its localization, a potential role in fission is supported also by functional studies. Protein ablation through a genetic knock-down and functional inhibition via dominant negative strategy show that in the absence of active *Tg*DrpC the mitochondria of daughter cells remain permanently interconnected. Strikingly, when using a dominant-negative form of *Tg*DrpC, which has an inactive GTPase domain and is tagged with YFP, we can see that the interconnections between mitochondria are decorated with the inactive *Tg*DrpC.

In both strategies, we observe a growth defect, suggesting that inhibition of *Tg*DrpC function is lethal in *T*. *gondii*. Similarly, defects in mitochondrial fission are lethal during embryo development in mice and humans [60, 61], and Drp1 has been involved in cancer development, as reviewed in [62]. In contrast, Dnm1 depletion is not lethal in yeast: in absence of fission, the mitochondrial network is only shaped by fusion, and as a result it becomes a long, tubular structure, which can still be transferred to buds during mitosis [17]. Although our analysis suggests that *Tg*DrpC is essential for mitochondrial fission, which subsequently leads to non-specific secondary defects while the parasite dies, we cannot exclude that *TgDrpC* has additional functions during parasite replication, as suggested in a recent study [63].

In this context, it is worth noting that *Tg*DrpC puncta are not only localised at mitochondria interconnections, but also at its periphery. This is in good accordance with previous observations showing that only a fraction of dynamin-related protein puncta on the MOM participates in mitochondrial division in yeast [64]. It was also observed that a different population of Dnm1 puncta, which is less dynamic than the one involved in mitochondrial fission, co-localises with the mitochondrial-ER-cortex anchor (MECA), a key element for the tethering of mitochondria, but the functional significance of this colocalisation is not yet clear [65, 66]. Previous work in Apicomplexa parasites has shown that the mitochondrion is in close juxtaposition with the IMC and suggested that the two organelles are tethered [5, 67]. It could be speculated that the puncta of *Tg*DrpC at the mitochondrial periphery are likewise involved in the formation of such tethers.

Another component of the fission machinery is the “adaptor complex” that brings the dynamin-related proteins Drp1/Dnm1 to the mitochondrial membrane. The composition of this complex varies in different organisms; in *T*. *gondii*, only one of the proteins involved is identifiable by BLAST, the transmembrane protein *Tg*Fis1, also conserved in *P*. *falciparum* [68]. We show here that this protein is not required for mitochondrial fission and is altogether dispensable for tachyzoite growth in culture. These findings are consistent to what observed in mammals, where Fis1 has only a minor role in mitochondrial fission, while other receptors, most notably Mff, Mid49 and MiD50, are required for recruitment of Drp1 and modulation of its GTPase activity [69]. Homologs of these proteins are not found in the *T*. *gondii* genome via BLAST, thus the question of how *Tg*DrpC is recruited to the mitochondrial membrane remains open.

Moreover, research has shown that Drp1/Dnm1 role in mitochondrial fission is facilitated by a “pre-constriction” step, which is mediated by two main actors: ER and actin. The ER wraps around mitochondria in sites of future fission [70] and in those sites actin polymerization occurs, mediated by the ER-localised formin 2 (INF2) and by the mitochondrial protein Spire1C [20, 71]. It is proposed that myosin II is subsequently recruited to these sites to help pre-constrict the mitochondrial tubules [72]. In contrast, no direct role of actin and myosins in mitochondrial fission has been observed in *T*. *gondii*, and *Tg*Act1 depletion does not affect mitochondria morphology [73]. Moreover, though ER and mitochondria seem to be strictly associated in *T*. *gondii*, we were not able to prove a direct interaction between their membranes during endodyogeny; better tools for the analysis of ER localisation and behaviour are required to further explore the role of the ER in *T*. *gondii* mitochondrial fission. It was recently shown that ER-mediated preconstriction is not the only mechanism that induces mitochondrial fission in mammalian cells, as other types of mechanical force can promote the same response [21]; thus, we cannot exclude that in *T*. *gondii* the preconstriction step is due to different mechanisms, such as the extension of the daughter bud’s IMC or the action of the basal body during cytokinesis, which have been proposed to be important for the fission of the apicoplast in this parasite [31, 74, 75].

In conclusion, we show here that the Apicomplexa-specific *Tg*DrpC is highly unusual among dynamin-related proteins, has only a conserved GTPase domain and is essential for mitochondrial biogenesis and for parasite survival. It therefore holds great potential as a drug target against apicomplexan parasites: future research on this point will benefit from the recent discovery of a novel class of compounds that directly inhibit Drp1 GTPase activity *in vitro* [76].

## Supporting information

S1 Fig Alignment of apicomplexan and other dynamin-related proteins. A) Sequence alignment of the N-terminus of *Tg*DrpC (aa 1–465) and the GTPase domain of human dynamin 3 (aa 16–304). (B) Clustal-Omega alignment of the indicated dynamin-related proteins. Comparison with orthologues in the Apicomplexa family shows that other 2 regions (red) are conserved in this group, but they do not correspond to canonical GED or Middle domain. Tg, *Toxoplasma gondii*; Ta, *Theileria annulata*; Bb, *Babesia bovis*; Pv, *Plasmodium vivax;* Pb, *Plasmodium berghei*; Cp, *Cryptosporidium parvum*. Black letters indicate identical and grey letters similar amino acids.
(TIF)

S2 Fig Induced TgDrpC knockdown at later time points leads to pleiotropic effects. (A) Plaque assay shows that, upon induction of rapamycin, the line *TgDrpC-*U1 shows a severe growth phenotype leading to collapse of parasites within the PV (upper lane and inset). Parasites were grown for 7 days on HFF cells in presence or absence of 50 nM Rapamycin. The experiment was performed in triplicate; representative images are shown. RH parasites were used as control. Scale bars = 200 μm. (B) Analysis of *TgDrpC-U1* after 96 hours of induction with rapamycin. Most vacuoles present “collapsed” mitochondria (αTom40); moreover, some of the bigger vacuoles look misshapen (as shown here with αGap45 staining) and in few cases the apicoplast (αHSP60) is not present in every parasite. Scale bars = 5 μm.
(TIF)

S3 Fig Overexpression of a wild-type copy of *Tg*DrpC does not affect parasite viability. (A) Schematics of the plasmid DD-GFP-DrpCwt_._ (B) Western blot of clonal *DD-GFP-DrpC*_*wt*_ parasites in presence and absence of Shield-1 using the indicated antibodies. (C) Plaque assay for *RH* and *DD-GFP-DrpC*_*wt*_ lines in presence and absence of Shield-1. The experiment was performed in triplicate; representative images are shown. Scale bars = 200 μm. (D) Immunoflorescence analysis showing DD-GFP-DrpCwt signal at 24 hours post-induction with Shield-1. (E) Time lapse analysis of parasites DD-GFP-DrpCwt/HSP60-RFP undergoing endodyogeny. (Scale bar = 5 μm. Red: HSP60-RFP; green: DD-GFP-DrpCwt)_._
(TIF)

S1 Video Time-lapse microscopy of TgDrpC-YFP/TGME49_215430-tdTomato expressing parasites (green and red respectively). The time lapse follows a vacuole with two parasites that just completed their cytokinesis but still show interconnected mitochondria until the connection is no longer detectable.
(AVI)
