# Supplementary figures and images for: A unique dynamin-related protein is essential for mitochondrial fission in Toxoplasma gondii
Source: PLoS Pathog. 2019 Apr 4;15(4):e1007512. doi: 10.1371/journal.ppat.1007512 (PMC6448817; doi:10.1371/journal.ppat.1007512)

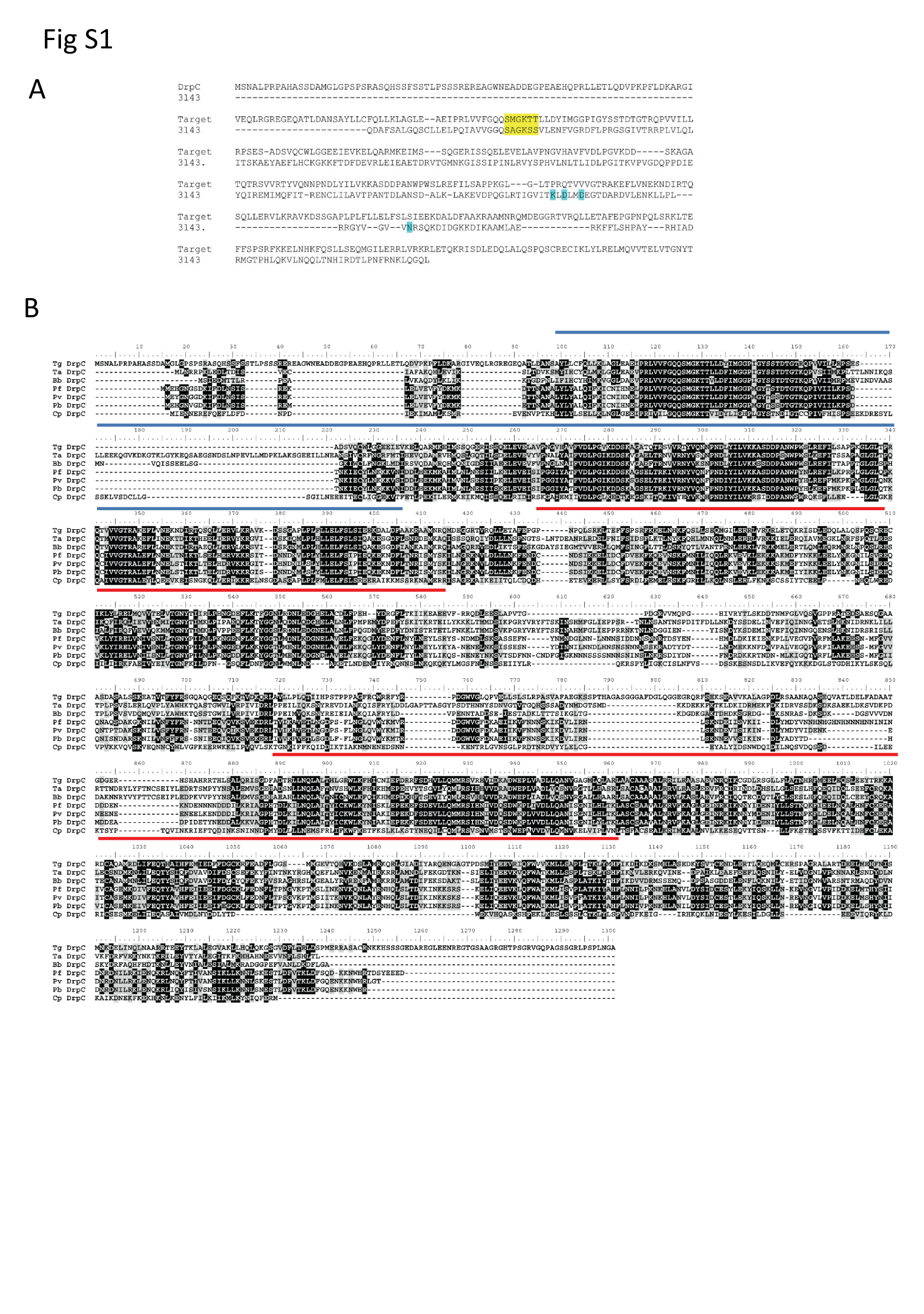

Supplement: S1 Fig — A) Sequence alignment of the N-terminus of TgDrpC (aa 1–465) and the GTPase domain of human dynamin 3 (aa 16–304). (B) Clustal-Omega alignment of the indicated dynamin-related proteins. Comparison with orthologues in the Apicomplexa family shows that other 2 regions (red) are conserved in this group, but they do not correspond to canonical GED or Middle domain. Tg, Toxoplasma gondii; Ta, Theileria annulata; Bb, Babesia bovis; Pv, Plasmodium vivax; Pb, Plasmodium berghei; Cp, Cryptosporidium parvum. Black letters indicate identical and grey letters similar amino acids. (TIF) [file ppat.1007512.s001.tif]

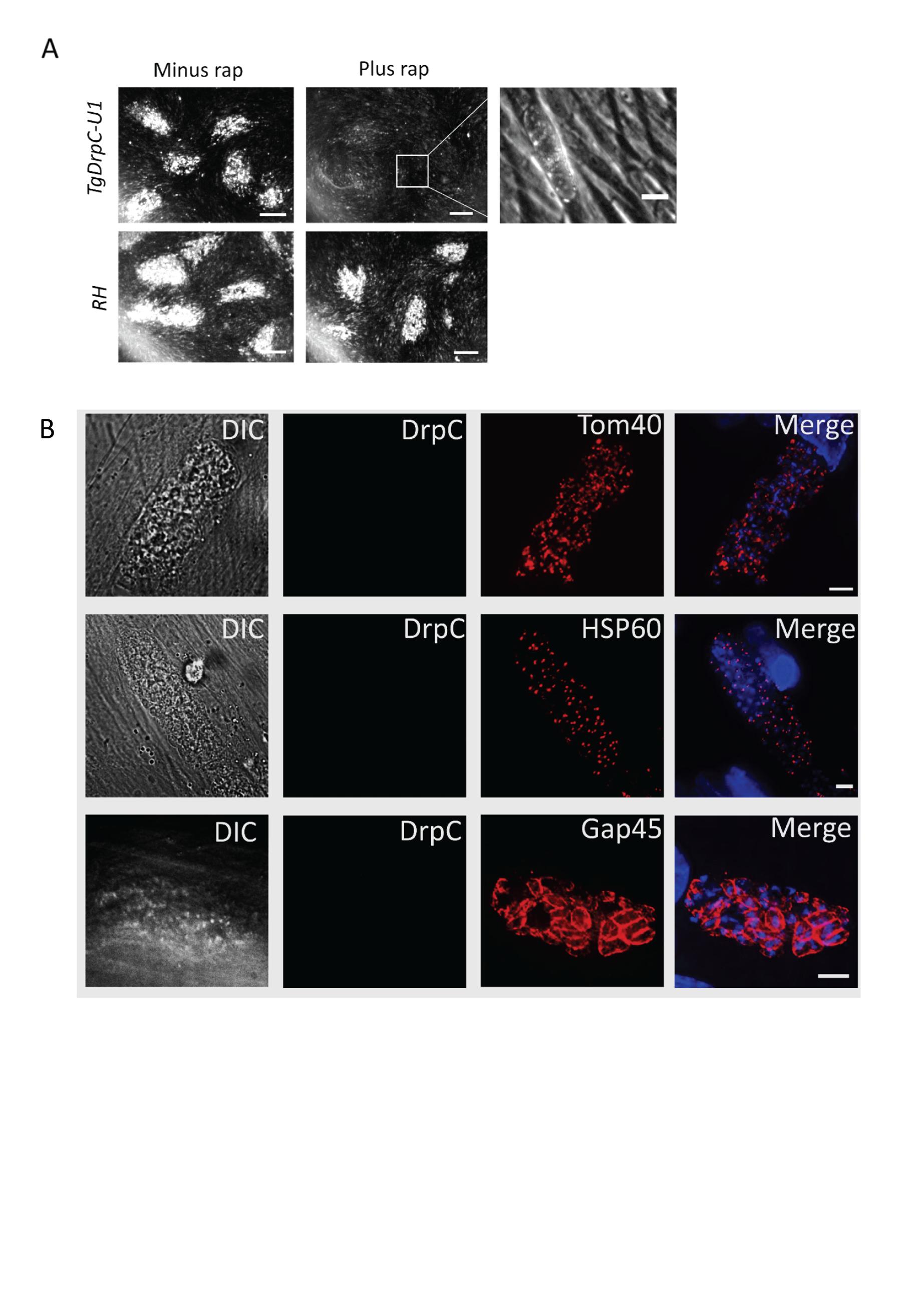

Supplement: S2 Fig — (A) Plaque assay shows that, upon induction of rapamycin, the line TgDrpC-U1 shows a severe growth phenotype leading to collapse of parasites within the PV (upper lane and inset). Parasites were grown for 7 days on HFF cells in presence or absence of 50 nM Rapamycin. The experiment was performed in triplicate; representative images are shown. RH parasites were used as control. Scale bars = 200 μm. (B) Analysis of TgDrpC-U1 after 96 hours of induction with rapamycin. Most vacuoles present “collapsed” mitochondria (αTom40); moreover, some of the bigger vacuoles look misshapen (as shown here with αGap45 staining) and in few cases the apicoplast (αHSP60) is not present in every parasite. Scale bars = 5 μm. (TIF) [file ppat.1007512.s002.tif]

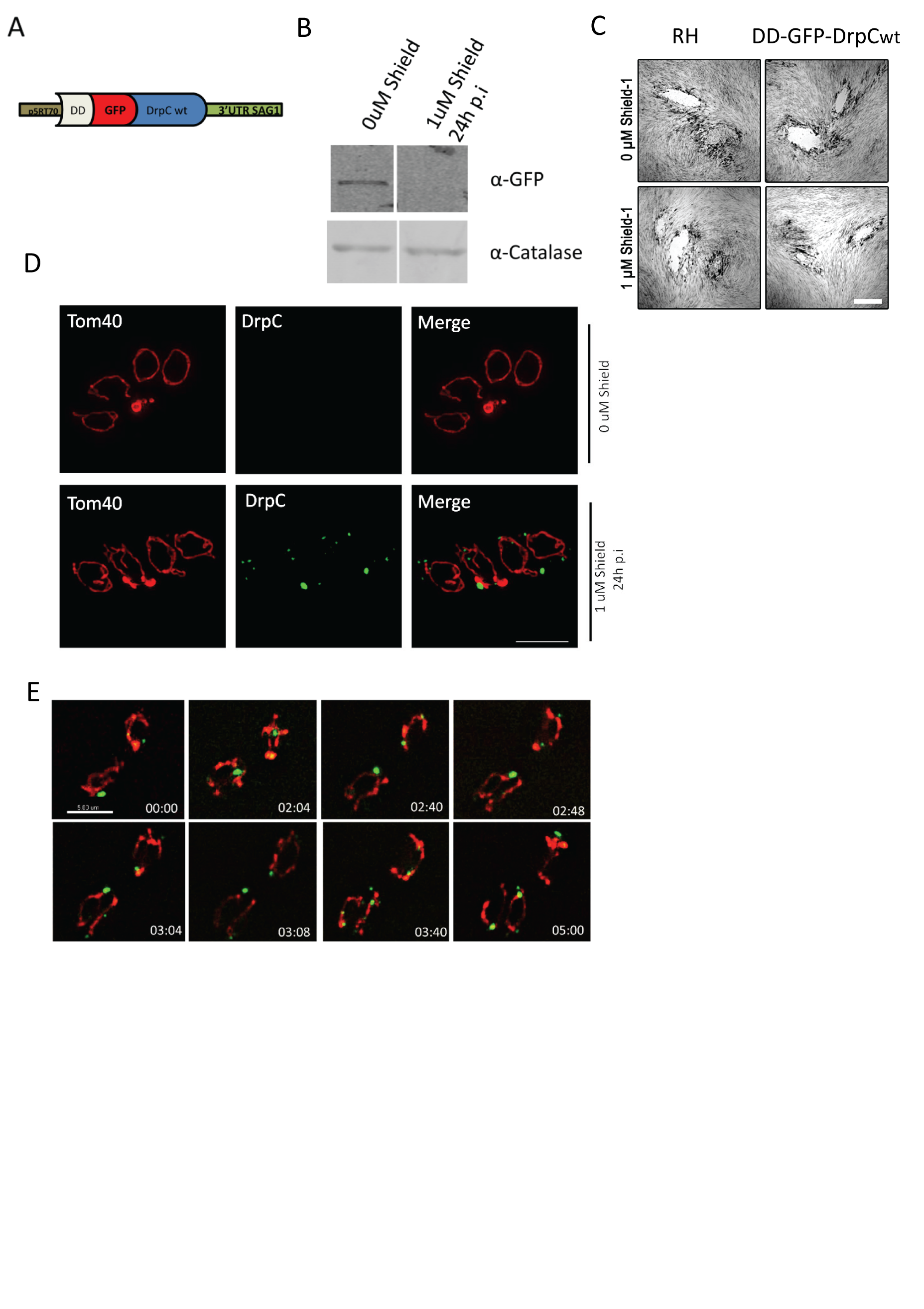

Supplement: S3 Fig — (A) Schematics of the plasmid DD-GFP-DrpCwt. (B) Western blot of clonal DD-GFP-DrpCwt parasites in presence and absence of Shield-1 using the indicated antibodies. (C) Plaque assay for RH and DD-GFP-DrpCwt lines in presence and absence of Shield-1. The experiment was performed in triplicate; representative images are shown. Scale bars = 200 μm. (D) Immunoflorescence analysis showing DD-GFP-DrpCwt signal at 24 hours post-induction with Shield-1. (E) Time lapse analysis of parasites DD-GFP-DrpCwt/HSP60-RFP undergoing endodyogeny. (Scale bar = 5 μm. Red: HSP60-RFP; green: DD-GFP-DrpCwt). (TIF) [file ppat.1007512.s003.tif]
